# The Superior Colliculus and Amygdala Support Evaluation of Face Trait in Blindsight

**DOI:** 10.3389/fneur.2020.00769

**Published:** 2020-07-17

**Authors:** Sara Ajina, Miriam Pollard, Holly Bridge

**Affiliations:** ^1^Department of Neurorehabilitation and Therapy Services, The National Hospital for Neurology and Neurosurgery, Queen Square, London, United Kingdom; ^2^Wellcome Centre for Integrative Neuroimaging, FMRIB, Nuffield Department of Clinical Neurosciences, University of Oxford, Oxford, United Kingdom; ^3^Institute of Neurology, University College London, London, United Kingdom

**Keywords:** blindsight, affective blindsight, hemianopia, cortical blindness, superior colliculus, amygdala, neuroplasticity, subcortical pathways

## Abstract

Humans can respond rapidly to viewed expressions of fear, even in the absence of conscious awareness. This is demonstrated using visual masking paradigms in healthy individuals and in patients with cortical blindness due to damage to the primary visual cortex (V1) - so called affective blindsight. Humans have also been shown to implicitly process facial expressions representing important social dimensions. Two major axes, dominance and trustworthiness, are proposed to characterize the social dimensions of face evaluation. The processing of both types of implicit stimuli is believed to occur via similar subcortical pathways involving the amygdala. However, we do not know whether unconscious processing of more subtle expressions of facial traits can occur in blindsight, and if so, how. To test this, we studied 13 patients with unilateral V1 damage and visual field loss. We assessed their ability to detect and discriminate faces that had been manipulated along two orthogonal axes of trustworthiness and dominance to generate five trait levels inside the blind visual field: dominant, submissive, trustworthy, untrustworthy, and neutral. We compared neural activity and functional connectivity in patients classified as blindsight positive or negative for these stimuli. We found that dominant faces were most likely to be detected above chance, with individuals demonstrating unique interactions between performance and face trait. Only patients with blindsight (*n* = 8) showed significant preference in the superior colliculus and amygdala for face traits in the blind visual field, and a critical functional connection between the amygdala and superior colliculus in the damaged hemisphere. We also found a significant correlation between behavioral performance and fMRI activity in the amygdala and lateral geniculate nucleus across all participants. Our findings confirm that affective blindsight involving the superior colliculus and amygdala extends to the processing of socially salient but emotionally neutral facial expressions when V1 is damaged. This pathway is distinct from that which supports motion blindsight, as both types of blindsight can exist in the absence of the other with corresponding patterns of residual connectivity.

## Introduction

Cortical blindness is the loss of sight following damage to the primary visual pathway in the brain. When damage occurs in one cerebral hemisphere, as is common in stroke, it causes homonymous hemianopia; a visual loss on the opposite side of the brain that is challenging to rehabilitate (1). However, patients with cortical blindness can demonstrate residual visual function via “blindsight,” which is an ability to respond to images in the blind field despite no conscious awareness of seeing anything at all (2). When the primary visual cortex (V1) is damaged, blindsight is believed to occur via intact subcortical structures and their direct connections to the non-striate visual cortex.

Highly salient emotional expressions are well-known to influence unconscious processing in blindsight and cognitive masking paradigms (3–6). However, the processing of additional facial expressions such as trustworthiness, gender, age, and personal identity is widely considered too subtle and complex to survive processing when V1 is damaged (7, 8). In fact, socially significant but emotionally neutral facial expressions are also believed to undergo rapid preconscious evaluation including trust, competence, and friendliness. Two major axes, trustworthiness and dominance, have been proposed to characterize the social dimensions of face evaluation (9) which is believed to represent a rapid adaptive mechanism for approach/avoidance behaviors in the perceiver (10).

It is possible that both implicit emotional processing and the evaluation of social traits may occur via similar underlying mechanisms involving the amygdala and its subcortical connections (10–12). If this is correct, we predict that patients with V1 damage and cortical blindness sustained in adulthood will demonstrate blindsight for faces exhibiting traits of trustworthiness and dominance, associated with an intact response in the amygdala and superior colliculus. We also anticipate that blindsight function will depend upon the underlying visual structures. Therefore, patients demonstrating blindsight for trustworthiness and dominance traits here may not necessarily have shown motion blindsight on previous studies and vice versa (13–15). This has potential implications for clinical practice, as blindsight may offer a mechanism for training and rehabilitation after V1 damage. If patients possess multiple targets to support training of different aspects of vision, this may further optimize the potential for recovery.

We used a set of emotionally neutral face images manipulated along two orthogonal axes of trustworthiness and dominance to generate five trait levels: dominant, submissive, trustworthy, untrustworthy, and neutral. We measured the ability of patients to detect and discriminate face traits significantly above chance using two 2-Alternate Forced Choice (2-AFC) experiments and compared fMRI responses in the blind and sighted fields of patients with and without blindsight. We predict that patients with unilateral V1 damage will demonstrate blindsight for these emotionally neutral face traits, which will be mediated by critical subcortical structures, including the amygdala and superior colliculus.

## Materials and Methods

### Participants

**Figure 1:**
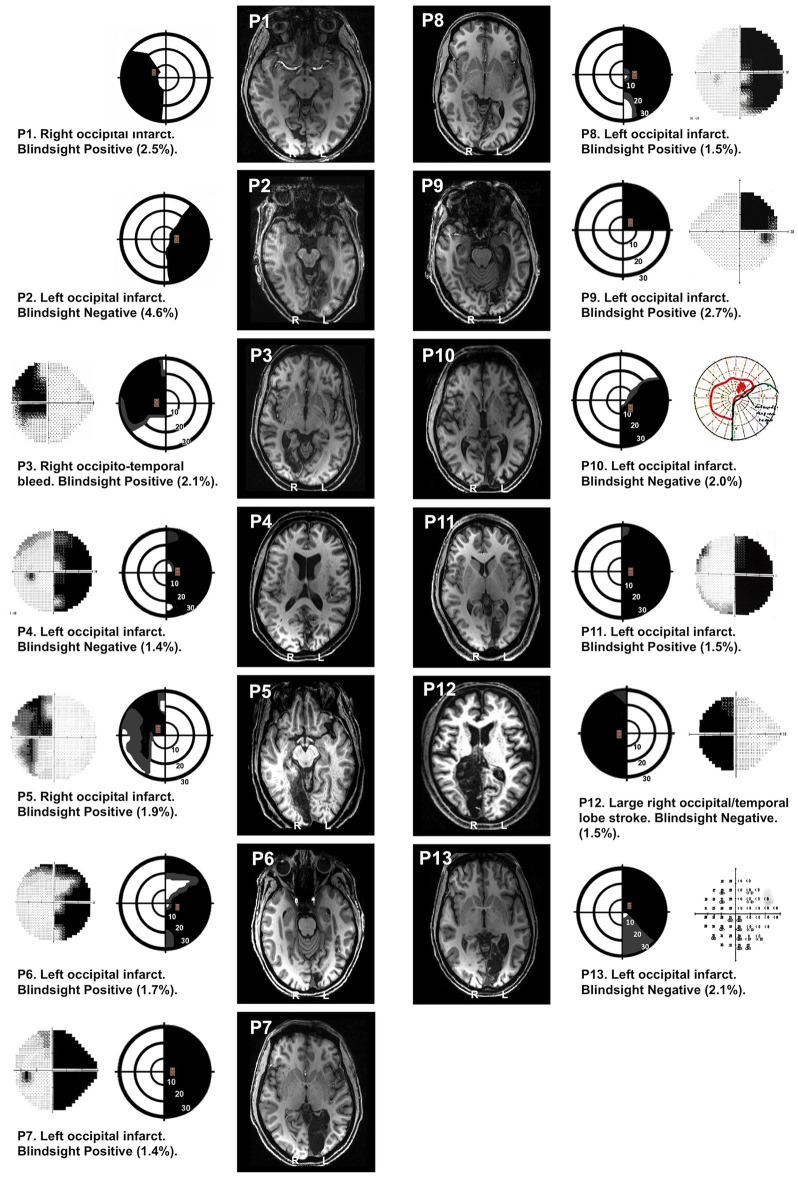
Visual field loss and T1 structural images. In each patient, perimetry reports are depicted schematically showing the location of target stimuli. Dense visual field loss is shown in black (<0.5%) and partial loss in gray (<2%). Stimuli were restricted to a region of dense visual field loss, a minimum of 2.5 degrees from fixation. Concentric rings represent increments in retinal position of 10 degrees, spanning the central 30 degrees. Equivalent perimetry data (Humphrey 30:2 except P10, who has Goldmann) are shown alongside (outer columns) where available. Blindsight status and estimates of the percentage of scotoma covered by the stimulus (%) are provided for each patient. Representative T1 structural axial slices demonstrate the lesion location, using radiological convention.

Thirteen patients with adult-onset unilateral V1 damage and corresponding visual field loss took part in the study (see Figure 1 and Table 1 for details). Average age at the time of participation was 52.2 years ± 14.3 SD; average time after pathology onset was 35.6 months (6–156 months). Written informed consent was obtained from all participants, and ethical approval was provided by the oxford research ethics committee (ref B08/H0605/156). While all participants participated in the MRI study, two participants (P5, P10) did not carry out experiment 1, and two participants (P1, P2) did not complete experiment 2 due to time constraints. All participants completed at least one behavioral experiment.

**Table 1:** Pathology location and patient demographics.

**Patient**	**Age range**	**Pathology**	**Time since lesion (M)**	**Visual field deficit**	**Blindsight status**
P1	26–30	Small cortical infarct in the striate cortex of the right occipital lobe, right PCA territory	13	LHH	Positive
P2	66–70	Left occipital infarct involving lingual gyrus and small portion of white matter	8	RHH	Negative
P3	66–70	Right occipito-temporal hemorrhage mostly restricted to right lingual gyrus	6	LHH	Positive
P4	41–45	Left occipital infarct with damage restricted to gray matter in the medial portion of the left occipital lobe	7	RHH	Negative
P5	66–70	Right occipital infarct with damage mostly restricted to the right lingual gyrus, including a small portion of white matter	16	LHH	Positive
P6	56–60	Left occipital infarct involving medial occipital cortex and extension to the left cerebellum	18	RHH	Positive
P7	46–50	Large iatrogenic left occipital infarct encompassing medial and ventral occipital cortex, with extension into the temporal lobe but sparing dorsolateral regions	84	RHH	Positive
P8	36–40	Left occipital infarct with damage isolated to the medial aspect of the left occipital lobe	7/16	RHH	Positive
P9	71–75	Left occipital infarct involving cortex and white matter in the medial left temporal and occipital lobes	19	RUQ	Positive
P10	56–60	Left occipital infarct with damage restricted to the posterior-medial left occipital lobe	96	RHH	Negative
P11	31–35	Left occipital infarct with damage restricted to the medial portion of the left occipital lobe	156	RHH	Positive
P12	56–60	Large right occipital/temporal lobe stroke	46	LHH	Negative
P13	41–45	Left occipital infarct involving medial and inferior portions of the left occipital lobe	6	RHH	Negative

*Descriptions include pathology nature and anatomical location, age range at participation in the study, time since pathology onset (months), visual field deficit, and blindsight status. M, months; HH, homonymous hemianopia; L, left; R, right; UQ, upper quadrantanopia*.

### Stimuli

**Figure 2:**
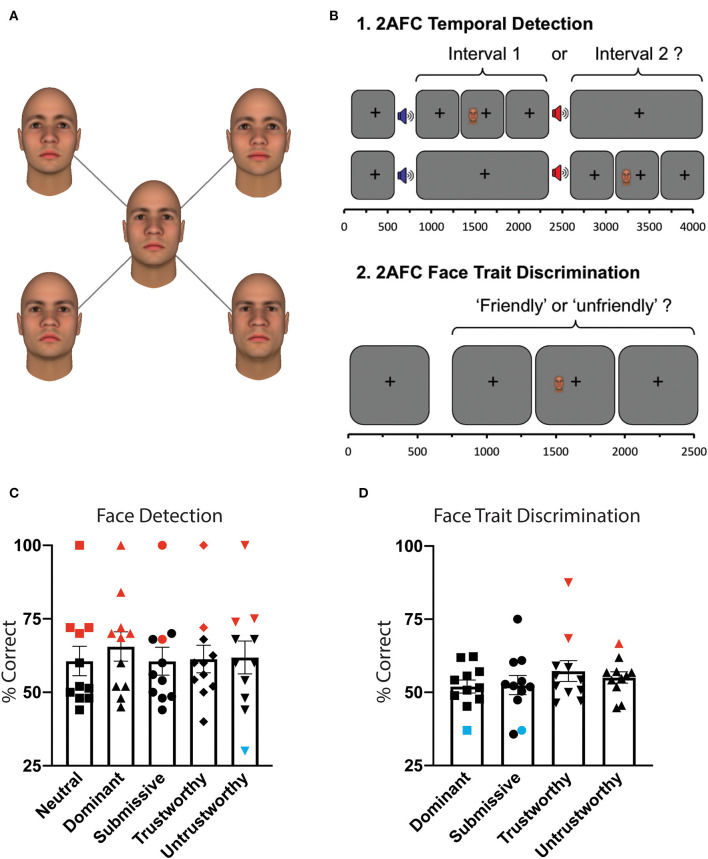
Behavioral paradigm and results. **(A)** Visual stimuli. All images represent one face identity, showing five levels of facial traits. Dominance and trustworthiness axes were manipulated in three steps (−3, 0, 3) of one standard deviation straddling the neutral. Faces were generated using the Facegen Modeler program (http://facegen.com version 3.1), based upon an extensively validated model (9). **(B)** Experiment 1: 2AFC temporal detection. Patients fixated on a central cross, with onset of each 1,500 ms interval alerted by a low (interval 1) or high pitch (interval 2) tone. Stimuli were located inside the scotoma (see Figure 1), and could appear in either interval at random, for a period of 500 ms. At the end of the trial, participants had to decide in which interval it appeared. Stimuli consisted of a face of 5.25 or 7.25° height, exhibiting either a dominant, submissive, trustworthy, untrustworthy, or neutral expression, at random. Experiment 2: 2AFC face trait discrimination. Throughout each trial of 2,500 ms duration, participants were required to fixate on a central black cross. During this time, the stimulus appeared inside the scotoma for 500 ms with jittered onset. At the end of the trial, patients had to indicate whether the face had been “friendly” or “unfriendly.” If they saw nothing, they were instructed to guess. **(C)** Mean behavioral performance for all participants who completed this task (*n* = 11) ± standard error of the mean (SEM) for 2AFC temporal detection, as a function of face trait. Statistically significant performance above chance was shown with red symbols, below chance in blue, all others in black. **(D)** Mean behavioral performance for all participants who completed this task (*n* = 11) ± SEM for 2AFC face trait discrimination.

Face stimuli were color images of emotionally neutral Caucasian faces taken from the trustworthiness and dominance datasets of the Social Cognition and Neuroscience Lab, Princeton University. Faces had been previously generated for another study (12) using the Facegen Modeler program (http://facegen.com, version 3.1). This customized software version provides two orthogonal parameters that allowed us to manipulate perceived trustworthiness and dominance based on an extensively validated model (9). We systematically varied the trustworthiness and dominance of the same face identity in seven steps (−3, −2, −1, 0, 1, 2, 3) of one standard deviation straddling neutral – see Figure 2A. The result was a set of 49 faces covering all possible combinations of trust and dominance in the employed range. Faces covered an ellipsoid area subtending either 5.25 or 7.25° (height), displayed on a uniform gray background of luminance 50 cdm^2^. Visual stimuli were presented using MATLAB (Mathworks) and the Psychophysics Toolbox (16, 17).

Stimulus location was restricted to the scotoma and its corresponding location in the sighted hemifield, a minimum of 2.5° from fixation. To select the precise location, we required a perimetry threshold *p* < 0.005 or < −20dB (whichever was more stringent) for pattern deviation compared to age-matched controls at the stimulus location. This meant that the patients in our study were unable to see even the brightest, unattenuated stimuli at that location in the visual field. Stimulus location had to be restricted to the boundaries of the fMRI display, which subtended 23° horizontally and 13° vertically. This influenced whether a 5.25 or 7.25° height stimulus was chosen, as the stimulus had to stay inside the “blind” field while remaining on screen. The stimulus of choice was 7.25°, but if this was not possible, the stimulus was reduced to 5.25°. The extent to which stimuli covered the scotoma (as a percentage) was estimated for each patient from the Perimetry Visual Field Index (VFI), provided in Figure 1. Stimuli in blindsight negative patients were no deeper into the visual field than blindsight positive patients (stimulus edge 4.1° ± 1.4 SD in blindsight positive, vs. 3.6° ± 0.8 SD in blindsight negative patients, *t* = 0.7, *p* = 0.5, df = 11).

### Behavioral Procedure

Outside the scanner, two behavioral experiments were performed: (1) 2AFC temporal face detection, and (2) 2AFC face trait discrimination (Figure 2B). The experiments were conducted on the same day as scanning, using a 60 Hz CRT monitor at a viewing distance of 68 cm. Face stimuli were identical to those used in fMRI testing, except that five identities were used for each trait, producing a set of 25 faces comprised of the five trait conditions. Throughout behavioral experiments, participants were asked to maintain fixation, with the investigator observing this in real-time using an Eyelink 1000 Eye Tracker (SR Research Limited, Ontario, Canada). Anyone making even a small eye movement into their damaged hemifield was given specific instruction not to do so, and it was explained that these data would have to be discarded.

At the start of the experiment, a test stimulus was used to confirm that patients were unable to see anything at the selected size and location in the visual field. This was done using a predicted aperture size and locus based upon prior perimetry results. If the patient was able to see any part of the test stimulus whilst fixating on the central cross, the aperture was repositioned 0.5° deeper into the scotoma (according to the Perimetry report) until the patient could no longer see any part of the stimulus at all. Any trials with eye position more than 1 degree from fixation were excluded from analysis.

Experiment 1: 2AFC Temporal Face Detection. Patients were asked to indicate whether a stimulus appeared in the first or second time-interval, using a two-alternate forced choice paradigm (Figure 2B). If they saw nothing, they were instructed to guess. Onset of each interval was indicated by a 500 ms auditory tone, 300 Hz marking onset of the first interval, and 1,200 Hz for the second. Visual stimuli appeared for 500 ms with jittered onset, while the participant fixated on a central black cross. The allocated interval (first or second) was generated at random. Face trait was altered parametrically between the five conditions at random, with an average of 28 trials per condition.

Experiment 2: 2AFC Face Trait Discrimination. Using only faces representing non-neutral traits (set of 20 faces), patients were asked to indicate whether each face that appeared was “friendly” or “unfriendly” (Figure 2B). If they saw nothing, they were instructed to guess. Visual stimuli appeared for 500 ms with jittered onset, whilst the participant fixated on a central black cross. Face trait was altered parametrically between the four trait conditions at random (neutral faces excluded), with an average of 42 trials per condition. Responses were counted as correct if submissive or trustworthy faces were classed as “friendly,” and dominant or untrustworthy faces were classed as “unfriendly.”

### fMRI Procedure

During fMRI scans, stimuli were presented on a 1,280 × 1,040 resolution monitor at the back of the scanner bore. Participants viewed stimuli via a double mirror mounted on the head coil. When in position, the screen subtended a visual angle of 23 × 13°. Each condition was presented separately to each hemifield, representing 10 randomly sequenced blocks. During each condition block, eight face identities were shown for 2 s each, with 0.5 s ISI. Rest periods of 10 s interspersed each condition. There were four runs in total, lasting 300 s each. Throughout the experiment (during condition and rest blocks), participants performed a task to maintain fixation by pressing a button every time a central fixation cross changed color from black to red. Color changes occurred at random, lasting 300 ms duration, and participants were instructed at the start to try not to miss any red crosses. It was emphasized that they must try to maintain fixation throughout and avoid moving their eyes around the screen. All participants scored over 80% on the fixation task averaged across all blocks (mean 94.1 ± 2.1% SE). An EyeLink 1000 eye tracker (SR Research Limited, Ontario, Canada) was used to confirm central fixation by recording eye position.

### Blindsight Definition

The presence or absence of residual visual function (blindsight) was determined according to the patient's ability to detect and/or discriminate the face stimuli above chance. This was defined as achieving either an average score, or a score for individual conditions that was significantly above chance, using a statistical threshold of *p* < 0.05 and a cumulative binomial distribution. This was an identical method to our previous work, except that the stimulus was a face rather than a drifting Gabor (15) or moving dots (13). The critical point was that patients performed above chance despite absent visual capacity in the targeted region of the visual field according to Perimetry (Figure 1). We ensured that a conservative threshold was used to target truly “blind” regions of the scotoma, which we demonstrated to be no different in patients with or without blindsight. Of note, we also observed two participants performed significantly *below* chance at discrimination of submissive/dominant faces, respectively (P9, P11). This was noteworthy, as it implied these participants could significantly discriminate between traits but could not correctly label the faces as “friendly” or “unfriendly” (instead, getting this the wrong way round). Indeed, both of these individuals performed above chance at detection and were therefore labeled as blindsight positive.

Using these criteria, eight patients were categorized as “blindsight positive,” as they could detect or discriminate the stimulus inside their blind hemifield significantly above chance (P1, P3, P5, P6, P7, P8, P9, P11). Of these individuals, four could also significantly discriminate “friendly” from “unfriendly” faces (P5, P7, P9, P11).

Of note, 11 of the patients (not P1 and P12) took part in previous studies (13, 15). P13 has been shown to be blindsight positive for motion but remained at chance in both experiments here and was therefore classified as “blindsight negative” for face traits. P7 was blindsight negative for motion but showed significant detection and discrimination above baseline for face traits and was therefore classified as “blindsight positive” here.

### Behavioral Eye-Tracker Results

Eye movements were defined as a change in fixation toward the scotoma of 1 degree or more. This would capture all eye movements irrespective of their type, i.e., saccadic, slow drift, nystagmus. The threshold of 1 degree ensured that stimuli could never be directly fixated, but would always remain inside the scotoma. Although micro-saccades were possible, these would not bring the visual stimulus into the seeing portion of the visual field. This methodology was the same as previous work (13–15). Six trials were removed from analysis in Experiment 1, and 11 trials from Experiment 2 due to eye movements of more than 1 degree toward the stimulus. This accounted for 0.46% of trials in Experiment 1, and 0.62% of trials in Experiment 2 that were excluded from analysis due to inappropriate eye position.

### fMRI Acquisition and Preprocessing

Scanning took place in a 3T Siemens Verio MRI scanner at the Functional Magnetic Resonance Imaging Center of the Brain (FMRIB, University of Oxford), using a 32-channel head-coil. Six hundred six functional volumes were acquired in a single session, duration 20 min (T2^*^-weighted EPI, 34 sequential 3 mm slices, repetition time = 2,000 ms, echo time = 30 ms, field of view = 192 mm). Magnetization was allowed to reach a steady state by discarding the first five volumes, an automated feature of the scanner. A high-resolution (1 × 1 × 1 mm voxels) whole head T1-weighted MPRAGE anatomical image (TE = 4.68 ms, TR = 2,040 ms, field of view = 200 mm, flip angle = 8 deg) and a field map with dual echo-time images (TE1 = 5.19 ms, TE2 = 7.65 ms, whole brain coverage, voxel size 2 × 2 × 2 mm) was also acquired for each participant.

Preprocessing and statistical analyses were carried out using tools from FSL (FMRIB's Software Library, www.fmrib.ox.ac.uk/fsl). Non-brain tissue was excluded from analysis using the Brain Extraction Tool [BET (18)], motion correction was carried out using MCFLIRT (19), images were corrected for distortion using field maps, spatial smoothing used a Gaussian kernel of FWHM 4 mm, and high-pass temporal filtering (Gaussian-weighted least-squares straight line fitting, with sigma = 13.0 s) was employed. Functional images were registered to high-resolution structural scans using FLIRT (20), and to a standard MNI brain template using FLIRT and FNIRT (21).

### ROI Analyses

The human amygdaloid complex is composed of several nuclei (22), however the limited spatial resolution of conventional fMRI makes it challenging to localize activity to any particular nucleus. Amygdala masks were defined using the Jülich Histological Atlas in FSL to combine the superficial, laterobasal, and centromedial nuclei groups. Lateral geniculate nucleus masks were Jülich-defined and superior colliculus masks were drawn manually in standard space, both being transformed to native space for ROI analyses using FNIRT and FLIRT. The average amygdala volume in patients measured 149 ± 17.5 SD voxels in the left, and 169 ± 13.7 SD voxels in the right; lateral geniculate nucleus measured 54.3 ± 13.0 SD in the left, 52.2 ± 4.0 SD in the right; superior colliculus measured 45.6 ± 8.2. There were no significant differences in ROI volumes between blindsight positive and negative patients.

Each of the 10 fMRI conditions (e.g., left hemifield, dominant trait) were entered into the general linear model as separate explanatory variables, and were contrasted against the baseline fixation task to generate 10 contrasts of parameter estimates (COPEs) for each condition in every voxel. Eight additional contrasts were generated by contrasting each face trait against neutral, within each hemifield, e.g., left hemifield, dominant > neutral. Signal change was then extracted from regions of interest within native space for each individual. The percentage signal change was calculated by scaling the COPE by the peak-peak height of the regressor and dividing by the mean over time. These measures were averaged across participants to generate group plots for signal change as a function of face trait and were used in all correlation analyses.

For an analysis of ROI co-variation over time, we used the residual timeseries in native space after stimulus responses had been regressed out. This provided a resting ROI1 vs. ROI2 correlation analysis for each participant, since task condition could have influenced intrinsic temporal correlations (23, 24). For completion, we also provided the correlation analysis for task-related activity.

### Whole Brain Group Analysis

Group analyses were performed to compare brain regions showing significant activation during blind hemifield stimulation between blindsight positive and blindsight negative patients. For evaluation of contralateral activation in the LGN, amygdala, and residual visual cortex it was necessary to align patient brains to a uniform pathological template, with lesions located in the same “left” hemisphere, corresponding to a “right-sided” visual deficit. This required that the structural and functional images of four patients (P1, P3, P5, P12) were flipped in the horizontal plane. The stimulus condition is therefore described as being presented to the “sighted ‘left' hemifield” or “blind ‘right' hemifield.”

All activation coordinates are reported in MNI space, and z statistic images are displayed on mean structural images for the group, which have been transformed to standard space. Mixed-effects analyses were carried out where multiple conditions were grouped together, e.g., sighted left hemifield, all traits vs. neutral; otherwise fixed effects analyses were used. A statistical threshold of *p* < 0.001 uncorrected was used to test for significance within the visual subcortex, for which we had *a priori* hypotheses. Elsewhere, correction for multiple comparisons was made using a cluster threshold of *p* < 0.01 (*z* > 3.1) unless otherwise stated. The reason for this dual approach was to allow the subcortical activations in the small structures such as the amygdala, LGN, and superior colliculus to be detected while preventing spurious activation in the cortex.

### Behavior fMRI Correlation

For correlation analyses between behavioral performance and fMRI activity, we used results from Experiment 1 (temporal detection task). Where this was not possible due to lack of data or performance at ceiling (*n* = 3), trait discrimination performance was used. This meant that data from all participants was included in the analysis. fMRI signal change in each ROI was derived from trait vs. neutral contrasts in native space, thus representing a preference for individual face traits over neutral.

## Results

### Dominant Faces Were Most Likely to Be Detected in the Blind Field

Detecting any face presented within the blind field was clearly a challenging task for the majority of participants, although one did perform at ceiling across all face categories. In total, five of the 13 participants were unable to detect any category of face significantly above chance in the blind hemifield (Figure 2C). Conversely, the other six participants who completed the detection task (*n* = 11) were able to detect at least one category of face (red markers). Furthermore, three of those able to detect faces (P7, P9, P11), along with a further patient who did not perform the detection task (P5), were also able to discriminate “friendly” submissive or trustworthy faces from “unfriendly” dominant or untrustworthy faces.

Detection was highest for dominant faces (mean detection 66.0% ± 5.0 SE, *n* = 11, *p* < 0.01, *t* = 3.2, df = 10). Across all participants, detection was significantly above chance for dominant, trustworthy (61.3% ± 4.7 SE, *p* = 0.04, *t* = 2.4, df = 10), and submissive faces (61.3% ± 4.6 SE, *p* = 0.03, *t* = 2.5, df = 10), but not untrustworthy (59.4% ± 5.9 SE, *p* = 0.14, *t* = 1.6, df = 10) or neutral faces (60.3% ± 5.1 SE, *p* = 0.07, *t* = 2.0, df = 10).

A repeated measure ANOVA did not find an effect of trait on detection [*F*_(40, 4)_ = 1.2, *p* = 0.3] or discrimination performance [*F*_(30, 3)_ = 0.3, *p* = 0.8]. However, a test of within subjects contrasts confirmed that detection of dominant faces was significantly higher than other traits [*F*_(10, 1)_ = 6.6, *p* = 0.03].

To assess for a correlation between discrimination and detection performance, we identified four blindsight positive participants (not at ceiling) who had carried out both experiments. Mean internal correlation was 0.2 ± 0.7 SD (range −0.70 to 0.73), confirming that an ability to detect a specific trait above chance was not associated with enhanced discrimination of that trait as being friendly or unfriendly. Indeed, the observation of instances of significant discrimination below chance suggests that such a correlation is likely to oversimplify unconscious residual visual abilities in these patients.

### Individuals Show Unique Interactions Between Performance and Face Trait

The relationship between performance and face trait varied considerably between participants. Five participants showed significant detection for dominant faces (P1, P3, P7, P8, P11), which was the largest number for an individual trait. One participant showed significant detection for only untrustworthy faces (P9, 75%), and another for trustworthy (72%) but not untrustworthy faces (54%, P8). In the discrimination experiment, performance was more varied. One participant (P5) could significantly discriminate trustworthy faces above chance (at 88%), while P7 could only discriminate negative traits (i.e., dominant and untrustworthy faces). Two participants showed significantly *reduced* discrimination below chance for submissive (P9) and dominant faces (P11, Figure 2D, blue symbols).

Participants also tended to show a difference between detection of positive vs. negative traits, although the direction of effect was not always consistent. The mean difference was 8.7% ± 1.9 SE, compared to a more moderate difference between dominance and trust axes (3.8% ± 1.3 SE). This meant that some participants were more likely to detect positive faces, while others were more likely to detect negative faces.

One participant with blindsight (P8) conducted two sessions of Experiment 1, 9 months apart. The relationship between detection and face trait in this individual was highly replicable over time (*R* = 0.94, *p* = 0.017). Together, this lends support to the hypothesis that, akin to the emergence from consciousness in experimental masking, the unconscious detection of faces may demonstrate individual differences and be influenced by participants' own personality traits of dominance and trustworthiness (12).

### Face Traits Elicited More Subcortical Activity Than Neutral Faces in Blindsight Positive Patients

**Figure 3:**
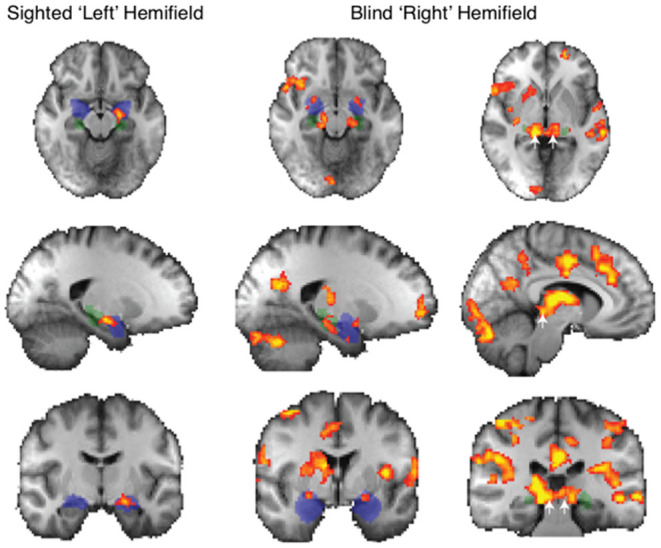
fMRI response to all face traits > neutral in patients with blindsight. Significant activity for face traits in the sighted field (left panel) and blind field (right panel, two columns). *Mixed-effects* analyses, *P* < 0.001 uncorrected for *a priori* regions of interest, elsewhere cluster corrected *p* < 0.01. Shaded blue areas are binarized Jülich-defined probabilistic maps of the amygdala, Jülich-defined LGN masks are green, and the superior colliculus is indicated by white arrows. *Z*-statistic range 2.3–4, radiological convention.

When faces were shown to the blind “right” hemifield, face traits averaged across all conditions caused significantly stronger subcortical activity than neutral faces in patients with blindsight. This included the bilateral amygdala, superior colliculus, and LGN (Figure 3, right panel). Amygdala (blue mask) preference for face traits was located in a region of the superficial nuclei group (peak MNI coordinates −20, 0, −12 on the left *z* = 3.1, and 24, 0, −10 right *z* = 3.2). There was an additional bilateral region at the border between the LGN (green mask) and superior colliculus (white arrows: *z* = 4.1 on right, coordinates 14, −32, −6; *z* = 3.6 on left, −12, −30, −6).

This widespread apparent activation contrasts starkly with the preference to these face traits when presented to the sighted “left' hemifield. In this case, only the amygdala showed a significant increase.

Blindsight negative patients did not show a preference for face traits vs. neutral faces in the blind hemifield, demonstrated using fixed- or mixed-effects analyses.

**Figure 4:**
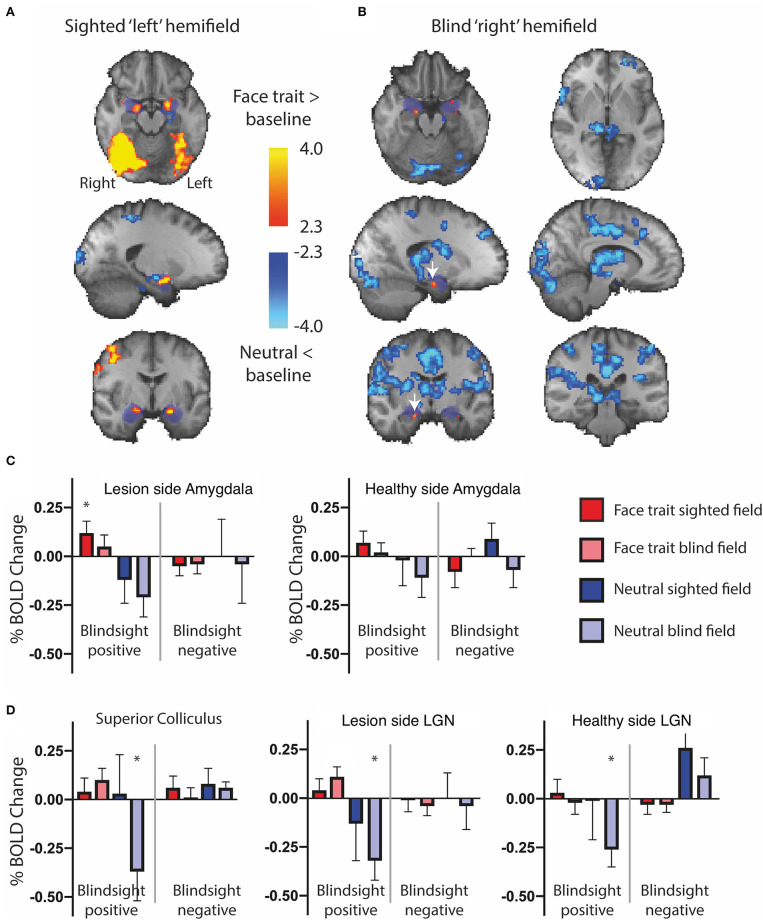
fMRI activity for face traits and neutral conditions compared to baseline in patients with blindsight. **(A)** Significant activity for face traits above baseline (red), and neutral faces below baseline (blue) driving the contrast in Figure 3, for the sighted “left” field, and **(B)** blind “right” field. *Mixed-effects* analyses, *P* < 0.001 uncorrected for *a priori* regions of interest, elsewhere cluster corrected *p* < 0.01. The significant cluster in the “right” amygdala is indicated by white arrows. Shaded blue areas are binarized Jülich-defined probabilistic maps of the amygdala. *Z*-statistic range 2.3–4, and −2.3–4, radiological convention. **(C)** ROI analysis of signal change for face traits and neutral faces compared to baseline in the amygdala, **(D)** superior colliculus, and LGN comparing blindsight positive (left panels) to blindsight negative (right panels) patients. Error bars represent SEM. Statistical symbol * represents a response significantly difference from 0 at 0.05 level for one sample *t*-test.

The widespread activation to the contrast of face traits vs. neutral faces in blindsight positive patients was driven slightly by increased activity for blind face trait conditions, but predominantly by the negative response to neutral faces shown in Figure 4. Here, the activation to face traits compared to baseline (mid-gray screen with fixation cross) is shown in red-yellow and the negative signal evoked by neutral faces compared to baseline is shown in blue. Presentation of face trait conditions to the sighted “left” hemifield (Figure 4A) evoked specific activity in ventral occipital regions, in addition to the amygdala bilaterally. In contrast, the reduction in BOLD signal in response to neutral faces is limited. When presented to the blind “right” hemifield (Figure 4B), the only region to be significantly activated above baseline in the whole brain analyses was the undamaged side of the amygdala (white arrow; *z* = 3.5, MNI 18, −10, −20).

Figure 4C shows the %BOLD change in the amygdala in both blindsight positive and negative patients. A 3-way ANOVA with blindsight status (negative or positive), blind or sighted field, and face trait or neutral as factors did not show any statistically significant main effects or interactions. Comparable data are shown for the superior colliculus and LGN in Figure 4D. Values significantly different from 0 are shown with an asterisk. Interestingly, the negative BOLD response appears to be specific to presentation in the blind hemifield in blindsight positive patients. Although noise from individual scans may have contributed to the negative BOLD, the exclusion of participants with the most sizeable and widespread signal below baseline (one out of eight patients, whole brain signal change one standard deviation below the mean) made no difference to the result.

### Dominant Faces Activated the Amygdala in Blindsight Positive Patients

**Figure 5:**
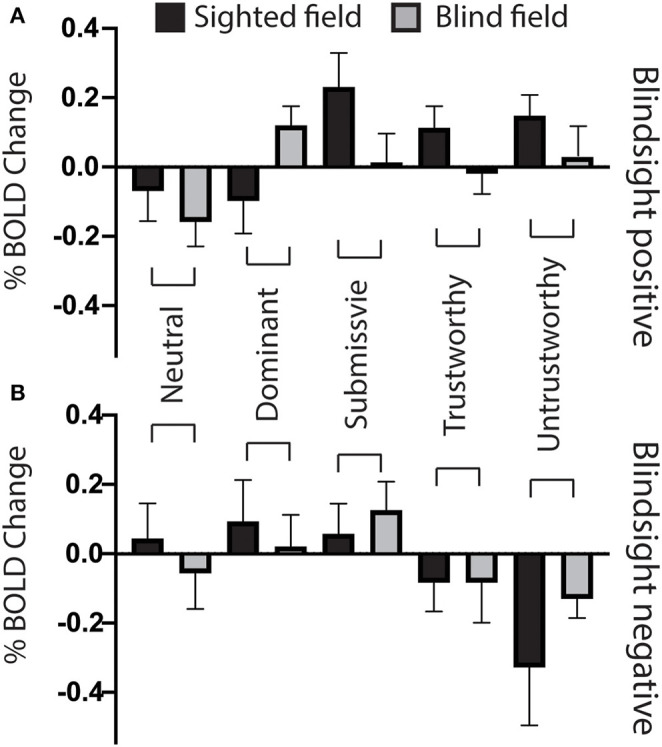
Bilateral amygdala ROI signal change for individual face traits compared to baseline. **(A)** Mean bilateral amygdala signal change as a function of face trait in blindsight positive patients, for the blind (gray) and sighted (black) fields compared to baseline. **(B)** Mean bilateral amygdala signal change for individual face traits in blindsight negative patients compared to baseline. Error bars represent SEM. Black columns are the sighted field, blind field in gray.

**Figure 6:**
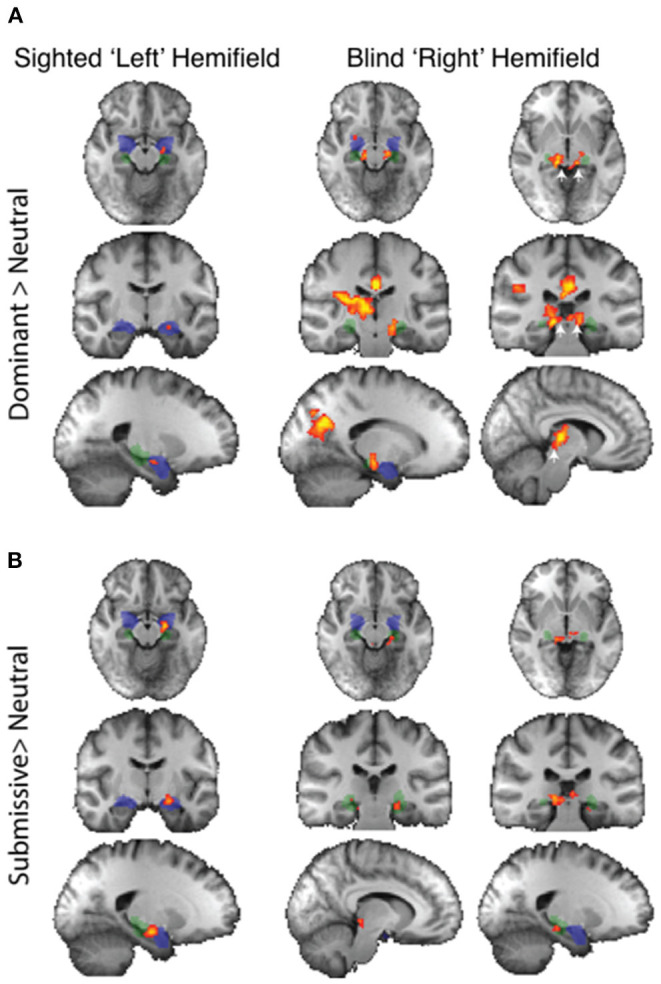
fMRI preference for dominance traits > neutral in patients with blindsight. **(A)** Upper panel shows activity for dominant faces > neutral in the sighted (left column) or blind fields (right two columns). **(B)** Lower panel shows equivalent activity for submissive faces > neutral. *Fixed-effects* analyses, *P* < 0.001 uncorrected for *a priori* regions of interest, elsewhere cluster corrected *p* < 0.01. Shaded blue areas are binarized Jülich-defined probabilistic maps of the amygdala, Jülich-defined LGN masks are green, and the superior colliculus is indicated by white arrows. *Z*-statistic range 2.3–4, radiological convention.

**Figure 7:**
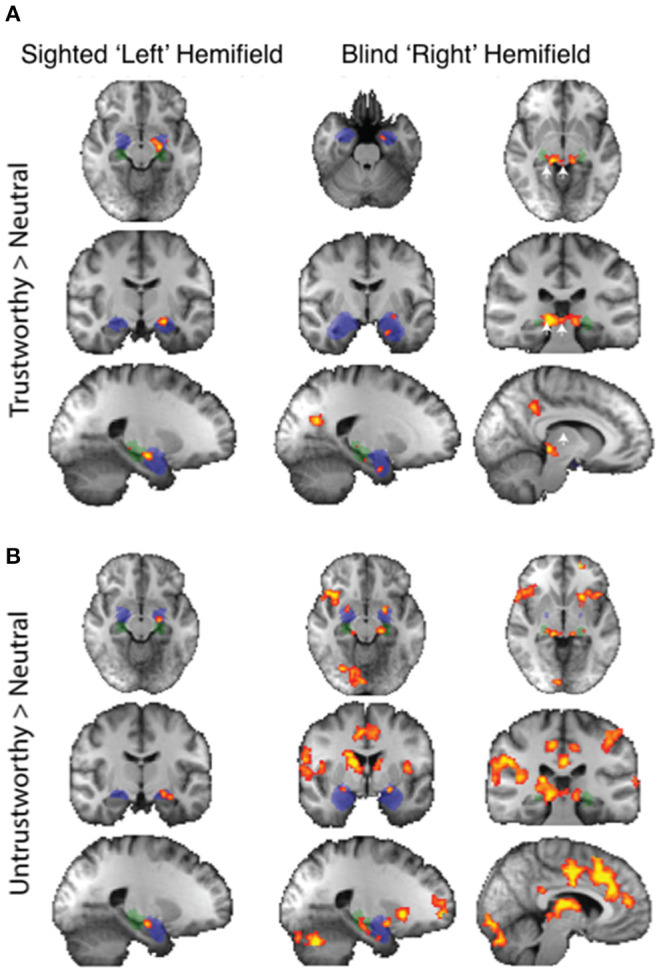
fMRI preference for trustworthiness traits > neutral in patients with blindsight. **(A)** Upper panel shows activity for trustworthy faces > neutral in the sighted (left column) or blind fields (right two columns). **(B)** Lower panel shows equivalent activity for untrustworthy faces > neutral. *Fixed-effects* analyses, *P* < 0.001 uncorrected for *a priori* regions of interest, elsewhere cluster corrected *p* < 0.01. Shaded blue areas are binarized Jülich-defined probabilistic maps of the amygdala, Jülich-defined LGN masks are green, and the superior colliculus is indicated by white arrows. *Z*-statistic range 2.3–4, radiological convention.

Regarding individual traits, since the amygdala is not lateralized to the visual field, the two sides were considered together. For the sighted field, the amygdala showed a significant interaction between blindsight status and traits on a repeated measure ANOVA [*F*_(4, 21)_ = 3.6, *p* = 0.01] (Figures 5A,B, black columns), although neither trait nor blindsight status were significant on their own (*F* = 1.4, *p* = 0.2, and *F* = 3.5, *p* = 0.07, respectively). In blindsight positive patients, only sighted submissive faces elicited ROI activity significantly above baseline (*p* = 0.04, *t* = 2.3, df = 15), with a large cluster demonstrating significant preference for submissive faces > neutral close to the LGN on contrast maps (Figure 6B, peak *z* = 3.4, MNI −20, −10, −14). Small clusters > 2.3 could also be seen exhibiting preference for sighted trustworthy (Figure 7A, peak *z* = 3.6, MNI −18, −10, −14) and untrustworthy traits (Figure 7B, peak *z* = 3.5, MNI −22, −12, −22) and, to a lesser extent, dominant traits (Figure 6A, *z* = 2.8, MNI −22, −10, −14), located in a similar region of the amygdala.

In the blind hemifield, the effect of individual traits compared to baseline was close to significance [*F*_(4, 21)_ = 2.3, *p* = 0.06). In blindsight positive patients, only dominant faces significantly activated the amygdala above baseline using ROI analyses (Figure 5A, gray columns, *p* = 0.049, *t* = 2.1, df = 15). Neutral faces, conversely, caused a significant reduction in activity below baseline (*p* = 0.04, *t* = 2.3, df = 15, shown in activity maps in Figure 4B). This means that contrasts of individual traits > neutral could have been significant despite activity for that trait being at or close to baseline. Trait contrast maps, however, showed a degree of variation in the location of peak activation to suggest this was not purely driven by negative signals for neutral faces (shown in Figures 6, 7). The dorsal left amygdala/LGN border region showed a significant preference for dominant over neutral faces (Figure 6A, *z* = 3.3, MNI −16, −18, −14), compared to the basolateral nuclei for trustworthy faces (Figure 7A, *z* = 3.2 MNI −20, −6, −26), and both the superficial group and a smaller cluster in the basolateral nuclei group for untrustworthy traits (Figure 7B, *z* =3.7, MNI −20,2,-12), the latter matching the coordinates for trustworthy traits (*z* = 3.0 MNI −18, −6, −26). No voxels in the amygdala showed preference for submissive traits over neutral, and the small cluster for trustworthy traits did not reach significance.

### Superior Colliculus Suppresses Response to Neutral Faces in Blindsight

**Figure 8:**
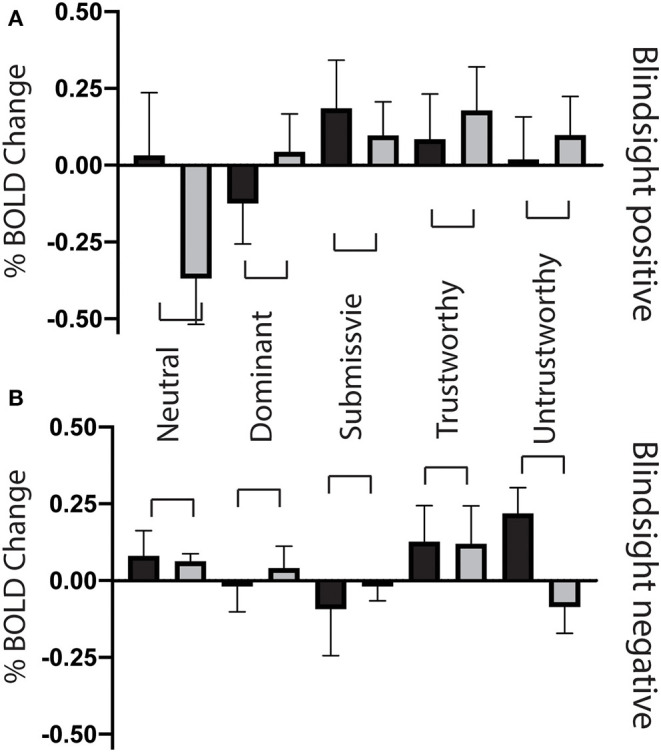
Superior colliculus ROI signal change for individual face traits compared to baseline. **(A)** Mean superior colliculus signal change as a function of face traits in blindsight positive patients, for the blind (gray) and sighted (black) fields compared to baseline. **(B)** Mean superior colliculus signal change for individual face traits in blindsight negative patients compared to baseline. Error bars represent SEM. Black columns are the sighted field, blind field in gray.

When faces were shown to the sighted field, individual traits had no effect on collicular activity [*F*_(4, 8)_ = 0.58, *p* = 0.7], and none of the individual conditions elicited significant activity above baseline (Figure 8, black columns). In comparison, in the blind field there was a weak interaction between blindsight status and individual traits [*F*_(4, 8)_ = 2.2, *p* = 0.088]. This was driven by a marked decrease in signals in blindsight positive patients when viewing neutral faces (*p* = 0.04, *t* = 2.2, df = 11).

**Figure 9:**
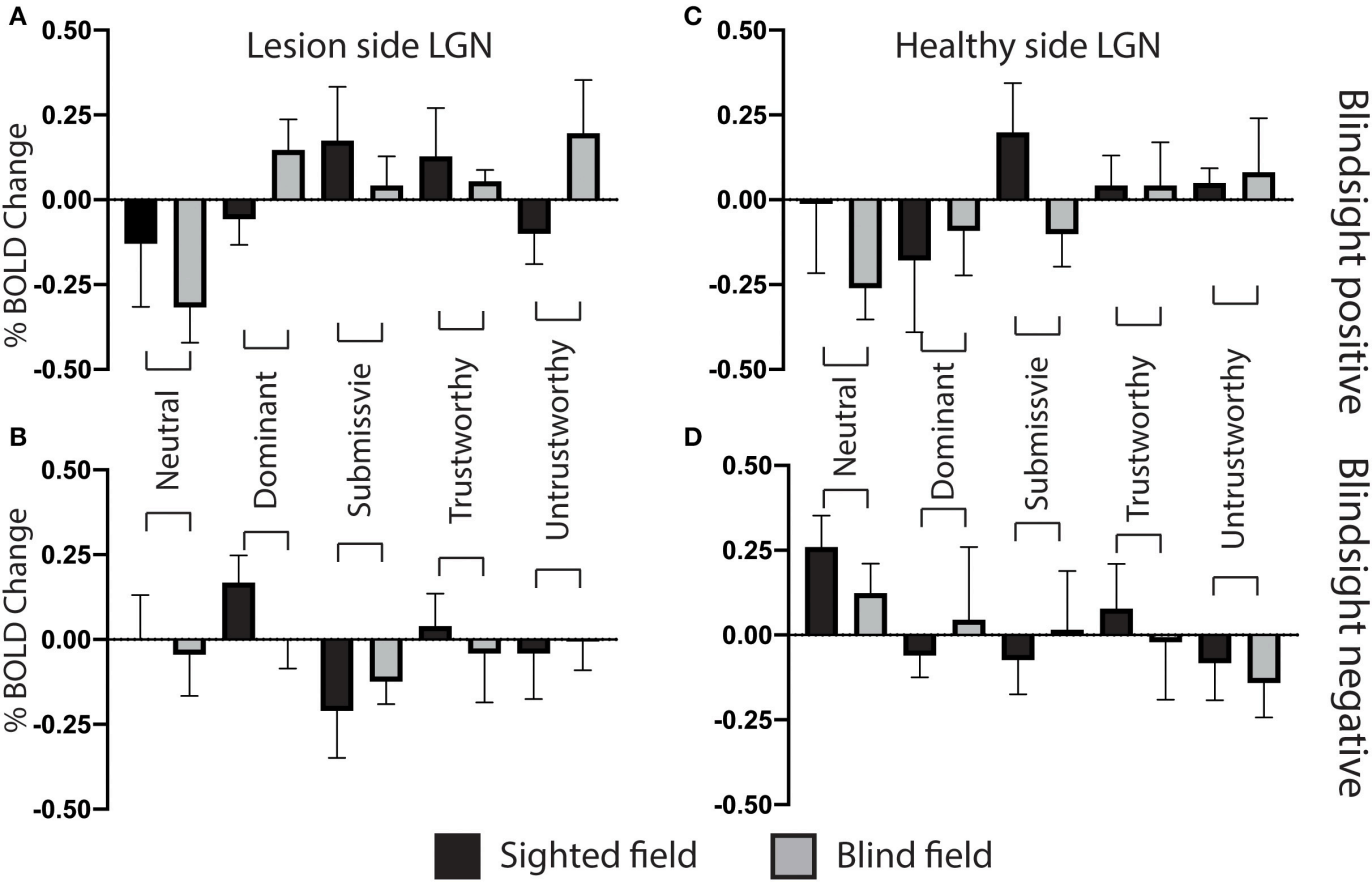
Lateral geniculate nucleus ROI signal change for individual face traits compared to baseline. **(A)** Mean lateral geniculate nucleus signal change on the lesion side as a function of face traits in blindsight positive patients, for the blind (gray) and sighted (black) fields compared to baseline. **(B)** Mean lateral geniculate nucleus signal change on the lesion side for individual face traits in blindsight negative patients compared to baseline. **(C)** Mean lateral geniculate nucleus signal change on the healthy side, blindsight positive patients. **(D)** Mean lateral geniculate nucleus signal change on the healthy side, blindsight negative patients compared to baseline.

In the contralateral LGN, a similar pattern was observed (Figure 9). There was no significant effect of individual traits in the sighted or blind hemifield [*F*_(4, 8)_ = 0.91, *p* = 0.5, *F*_(4, 8)_ = 1.9, *p* = 0.12, respectively], although activity in the blind field of blindsight positive patients was slightly less uniform for non-neutral traits than in the superior colliculus (Figure 9A, gray columns).

For the LGN and superior colliculus, the presence of a negative signal change for neutral faces and, to a lesser extent, activity above baseline for individual traits would have contributed to the marked contrast in brain maps for traits > neutral faces in the blind hemifield (Figures 6, 7). The change in response in blindsight positive patients may be reflective of the connectivity of recruited blindsight pathways. If, for example, blindsight was supported by a connection between the superior colliculus and amygdala, the superior colliculus may be more likely to share its response pattern in blindsight positive patients.

### Functional Connectivity Between the Superior Colliculus and Amygdala in the Damaged Hemisphere Was Critical for Blindsight

**Figure 10:**
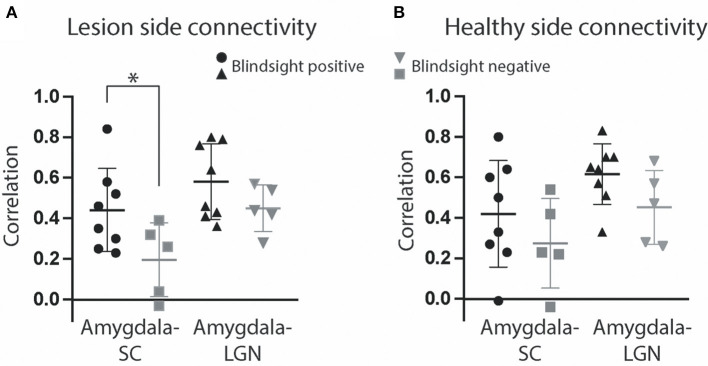
Functional connectivity of collicular subcortical pathways. **(A)** Mean correlation between the superior colliculus and amygdala (circle and square symbols represent results for individual participants) and amygdala and LGN (triangles) in the same lesioned hemisphere over the entire fMRI timeseries, after stimulus-evoked activity has been regressed out. **(B)** Correlation of the same regions of interest in the healthy hemisphere. Error bars represent SEM for correlation coefficients. Blindsight positive patients are shown in black, and blindsight negative patients are gray. Statistical symbol * represents significance at 0.05 level for two sample *t*-test.

In addition to the presence or absence of fMRI activity in blindsight, we were interested in whether there was a difference in the functional connectivity between subcortical nuclei. A functional connection between the amygdala and superior colliculus in the lesioned hemisphere was significantly different in patients with or without blindsight (Figure 10A, 0.44 ± 0.07 SE vs. 0.20 ± 0.08 SE, *t* = 2.2, *p* = 0.046, df = 11) using a time series analysis after stimulus-dependent activity had been regressed out. This was also true for an analysis of task-related activity (0.44 ± 0.07 SE vs. 0.16 ± 0.10 SE, *t* = 2.4, *p* = 0.038, df = 11). In contrast, there was no difference between blindsight positive or negative patients in connectivity between the damaged side LGN (ipsilesional) and superior colliculus (Figure 10A). Moreover, there were no differences in connectivity between these structures in the healthy side (Figure 10B). Functional connectivity between the damaged side of the amygdala and superior colliculus in blindsight negative patients was the only connection that was not significantly > zero (0.20 ± 0.08 SE, *t* = 2.5, *p* = 0.07, df = 4). Connectivity between the healthy side of the amygdala and superior colliculus was also close to zero (0.27 ± 0.10 SE, *t* = 2.7, *p* = 0.05, df = 4). This may reflect an involvement of the superior colliculus or its connections with damage in some of the blindsight negative patients (see Supplementary Figure 1).

### Amygdala and LGN Responses Correlated With Behavior in the Blind Field

**Figure 11:**
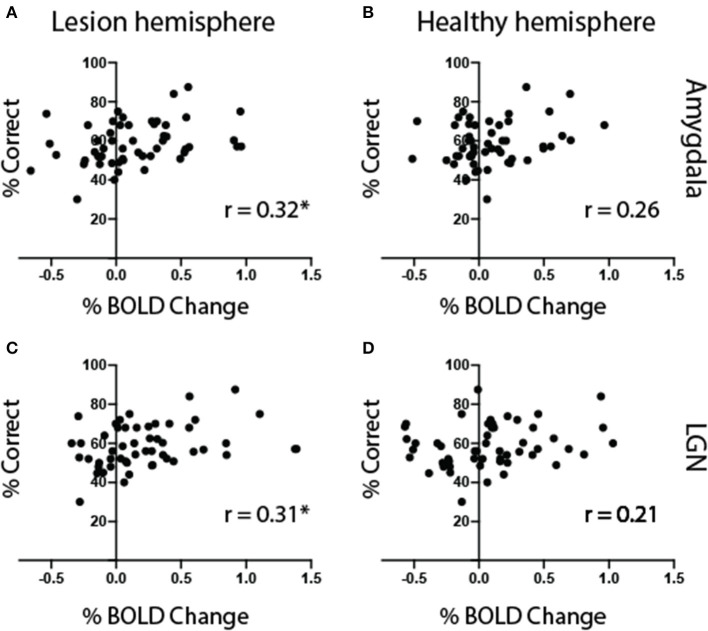
Amygdala and LGN activity correlates with behavior. Mean behavioral performance plotted against signal change in the amygdala **(A,B)** and LGN **(C,D)** for faces shown to the blind visual field of all patients (*n* = 13). The left column shows correlation with ROIs in the lesioned hemisphere, and the right column the right, healthy hemisphere. Symbols represent results for individual face trait conditions, in each participant. Statistical symbol * represents significant correlation at statistical level of 0.05.

The superior colliculus showed similar levels of activity for all non-neutral face traits in the blind field. In contrast, the amygdala showed notable difference–in particular, stronger activity for faces that were dominant. We were interested in whether the level of activity generated by a preference for individual face traits over neutral in the blind hemifield correlated with behavioral performance across all 13 participants. As expected, there was no association between performance and activity in the superior colliculus (*r* = 0.07, *p* = 0.6). The amygdala in the lesioned hemisphere, however, showed a significant correlation between signal change and performance (Figure 11A, *r* = 0.32, *p* = 0.02, but not in the healthy hemisphere Figure 11B). Interestingly, the LGN also showed a significant correlation with performance (Figure 11C, *r* = 0.31, *p* = 0.02, but not in the healthy hemisphere Figure 11D). Therefore, the greater the preference in the amygdala and LGN, the higher the chance for that face to influence behavior in patients with cortical blindness. This also suggested that the ROI group averages had not been sensitive to individual differences in activity. If, e.g., one participant had been most sensitive to dominant faces while another to untrustworthy faces, as implied from behavioral results, this would have been averaged out.

### Participants Exhibiting Distinct Blindsight Functions

The large number of patients in the current study permitted some crossover with previous investigations into blindsight. Two participants in particular, who took part in motion blindsight functional imaging experiments published previously (13, 25), showed distinct blindsight performance in the two domains. P13 was blindsight negative in the current study, as she performed at chance for both detection (mean 52.0% ± 8.9 SD) and trait discrimination (mean 48.5% ± 5.8 SD) tasks, including for individual traits. Conversely, she is blindsight positive for motion as she can detect high contrast drifting Gabors (15) and black moving dots significantly above chance (13). Previous studies found that a connection between motion area hMT+ and LGN was critical to blindsight and was preserved in this individual. In the current study, this participant showed absent functional connectivity between the superior colliculus and other subcortical structures (*r* = −0.04 with contralesional amygdala, *r* = −0.03 with ipsilesional amygdala, *r* = 0.05 with ipsilesional LGN). In contrast, the connection between the LGN and amygdala in her damaged hemisphere appeared intact (*r* = 0.42). Together this suggests that a posterior circulation stroke was able to spare connectivity between the LGN and hMT+ or the amygdala, while impairing connectivity between the superior colliculus and LGN or amygdala, thus resulting in distinct blindsight functions. When looking more closely at her structural T1 scans, there is the suggestion of an area of lower signal intensity in the vicinity of (but not adjacent to) the superior colliculus, in the damaged left hemisphere (Supplementary Figure 1). This highlights the difficulty of inferring damage from structural scans alone, and the importance of using additional measures such as functional connectivity or diffusion tractography.

The precise opposite was observed in participant P7, who was described as blindsight negative for motion but could detect (mean 72.1% ± 8 SD) and discriminate negative face traits (mean 61.9%) significantly above chance. While he previously demonstrated impaired connectivity between the LGN and hMT+, in the current study he showed normal connectivity between the superior colliculus and amygdala (*r* = 0.52 ipsilesional), superior colliculus and LGN (*r* = 0.75), and LGN and amygdala (*r* = 0.76), reflecting the location of his pathology close to hMT+ in the occipital lobe.

To summarize our results, blindsight was associated with significant amygdala activity for face traits in the blind hemifield, as well as suppression of the superior colliculus response to neutral faces. A functional connection between these structures in the damaged hemisphere was critical, with amygdala activity significantly correlating with behavioral performance across all participants. Together, this suggests that affective blindsight involving the superior colliculus and amygdala can extend to the processing of socially salient emotionally neutral faces when V1 is damaged. This pathway is distinct from those supporting motion blindsight, as both types of blindsight can exist in the absence of the other, with corresponding patterns of residual connectivity.

## Discussion

This is the first study to investigate the human response to socially salient but emotionally neutral faces in cortical blindness. These responses are believed to rely on similar pathways to those supporting unconscious emotional processing (5, 10). Affective blindsight is well-described in literature, and is often the topic of individual case studies, such as that of GY and TN (3–5, 26, 27). The current study measured fMRI responses to faces showing different expressions of trustworthiness and dominance in patients with unilateral V1 damage, along with their ability to detect and discriminate these face images in their blind field. These are traits that predominantly characterize the social dimensions of face evaluation (9). By comparing behavioral performance in the blind field with neural activity and functional connectivity in key regions of interest, we showed that the amygdala and its functional connection with the superior colliculus was critical for face trait blindsight.

### Blindsight Is Specific to Its Underlying Structures and Pathways

The amygdala, superior colliculus, and dorsal thalamus (5, 26–29) have all been shown to support affective blindsight, including connections with cortical somatosensory association areas (3). We observed bilateral amygdala preference for face traits in the blind field of blindsight positive patients, supporting our prediction that similar mechanisms may underlie unconscious face trait processing. In early studies, GY showed an ability to discriminate happy/sad, angry/sad, and angry/fearful faces above chance (4), and elicit similar bilateral amygdala responses for fearful and aversive-conditioned faces.

The superior colliculus is broadly implicated in blindsight. Monkeys retain saccadic eye movements toward a target in their blind field after a V1 lesion, but this ability and the potential for recovery is lost if the ipsilesional superior colliculus is also inactivated (30, 31). Similarly, superior colliculus neurons may retain their response to visual stimulation after striate cortex removal, although this response becomes considerably weaker (31–34). In human studies, a critical role has been suggested in motion blindsight after brain damage at birth or in early childhood (35–37), while the LGN appears critical for adult-onset cortical blindness (13, 15). There is also some evidence for superior colliculus involvement in affective blindsight (5, 28, 38).

Our evidence that the superior colliculus is critical for face trait blindsight was 3-fold. Firstly, we observed significant activity for blind hemifield responses to face traits contrasted against neutral faces in blindsight positive patients, which was absent in blindsight negative patients. This activity was due to a combination of negative BOLD activity in response to neutral faces and increased activity to face traits, a pattern that was only present in blindsight positive patients. Secondly, functional connectivity between the superior colliculus and amygdala in the damaged hemisphere was the only effective subcortical connection present in blindsight positive patients but not in blindsight negative patients. Thirdly, one patient who had no blindsight for face traits was shown previously to exhibit motion blindsight and an intact LGN-MT pathway in the damaged hemisphere. In the current study, she showed absent functional connectivity between the superior colliculus and other subcortical structures, despite intact connectivity between the LGN and amygdala. This is significant, as it suggests that blindsight is specific to its underlying structures and pathways, and has important functional implications for rehabilitation. The targeting of intact residual pathways may support useful changes in visual performance after sight loss caused by brain injury. Cortical blindness is notoriously persistent and resistant to treatment (1), but a subcortical pathway supporting functionally important affective and social processing could offer a potential mechanism for therapy.

Unlike the current study, fearful faces in the blind field of GY did not appear to elicit significant collicular activity (5). This could be because they employed a contrast of fearful vs. happy faces. We found that the superior colliculus, unlike the amygdala and lateral geniculate nucleus, showed similar activity for positive or negative valence faces. Perhaps the superior colliculus, therefore, responded to unseen happy expressions as well as negative expressions of threat–thus canceling out signal change.

Our result has also permitted a useful conclusion on motion blindsight. We can infer that the geniculate-extrastriate connection in motion blindsight was unlikely to be collicular in origin, as a functional connection between the superior colliculus and LGN appeared to be absent in one participant who previously demonstrated motion blindsight and an intact connection between LGN and hMT+. This had been postulated to account for disparate findings between well-known blindsight studies (30, 39), but is unlikely to be critically important.

### Implicit Social Dimensions of Face Trait

Socially significant facial expressions play an important role in rapid, and perhaps unconscious, evaluation of qualities such as trust, competence, and friendliness. Inferences may be extremely fast yet quantitatively replicable across time (40). It has been proposed that two major axes, trustworthiness and dominance, predominantly characterize the social dimensions of face evaluation (9). Threatening faces are believed to be both untrustworthy, signaling that a person may have harmful intentions, and dominant, signaling that a person is capable of causing harm (9). Such traits can be processed preconsciously, and differentially influence “time to emerge” in a monocular masking paradigm (12). Dominant and untrustworthy faces take longer to recognize than neutral faces, postulated to occur via a passive freezing response involving the amygdala, brainstem, and basal forebrain system (11, 12). It has been suggested that the amygdala has a critical role in processing implicit negative trait judgments, explained in terms of rapid adaptive mechanisms for approach/avoidance behaviors in the perceiver (11, 12, 41).

Highly salient emotional expressions are well-known to influence unconscious processing in masking and blindsight. However, the processing of additional facial expressions such as trustworthiness, gender, age, and personal identity is widely considered too subtle and complex to survive processing when V1 is damaged (7, 8). The current study has shown that unconscious processing of dominance and trustworthiness is possible after V1 damage, and is likely to be supported via the superior colliculus and its connection with the amygdala and/or lateral geniculate nucleus. The unconscious processing of such facial expressions could be considered an extension of emotional expression, despite being emotionally neutral (9). Indeed, judgments of face trustworthiness do reflect similarity of facial features to happy and angry expressions (40). Our results align well with the literature on face traits and emotional processing, both of which are believed to undergo preconscious, rapid processing to enable detection of threatening situations and promote survival (5, 42), although the effects are predictably weak given the variability of patients.

### Positive vs. Negative Facial Expressions

We found that positive and negative valence faces had similar levels of activity compared to neutral faces in the amygdala of blindsight positive patients, despite slightly greater activity for dominant faces. This may appear at odds with reports of amygdala-lesioned patients who show the greatest deficits in recognition of fearful faces relative to other emotions (43), leading to the theory that the amygdala is specialized for the processing of evolutionary-relevant expressions of threat (5, 42, 44, 45).

A possible explanation for this difference was that the visual processing in our study was unconscious. In implicit face trait processing, the amygdala has shown both a linear (negative) relationship with trustworthiness (46) and a U-shaped response for facial expressions including trustworthiness (10, 47) and attractiveness (48), where activation is stronger for faces at both extremes of the dimensions rather than at the middle. The amygdala is also postulated to experience a “specificity trade off” during unconscious processing, with insufficient capacity to focus purely on negative expressions (6).

### Individual Variability in Face Trait Processing

We observed wide variation in the ability of participants to detect different traits, perhaps even demonstrating suppression of certain faces below chance. This was highlighted by the observation of one participant, P8, whose unique performance was highly replicable over time (*R* = 0.94, *p* = 0.017). Healthy individuals have been shown to exhibit variability in the time it takes for masked faces to emerge into consciousness, according to their own personality traits (12). Using continuous flash suppression, researchers have shown that participants with more submissive and untrusting traits tended to suppress dominant faces longer, perhaps synonymous with a freezing preconscious fear-response that is greater in individuals who are more likely to take threat more seriously. It was hypothesized that this could be mediated via the amygdala, brainstem, and basal forebrain system (11), which would be consistent with the results found here.

### The Role of Negative Bold Signals

One of the most significant results in this study is the reduction of the BOLD signal in response to neutral faces presented in the blind field compared to a mid-gray background in blindsight positive patients. It is a pattern that is not present in patients without blindsight, and to a much lesser extent when stimuli are presented to the sighted field. The explanation for this pattern is not obvious, and indeed, the structures showing deactivation are those around subcortical visual areas such as the superior colliculus and pulvinar, in addition to the visual cortex.

There are previous observations of negative BOLD signal change during visual stimulation of the blind hemifield, including fMRI response to motion stimuli (13, 15, 25, 39). In ROIs with significant activation during certain blind conditions, lower salience images also appear to cause signal change to fall below zero. In these examples, critically, the pattern of fMRI activity is preserved despite this decrease. There are many possible reasons for signal change to drop below baseline (49). At its most simple, it requires that true “baseline” only represents activity that has occurred during rest blocks, which in turn requires that the hemodynamic response curve represents the underlying neurophysiology accurately. If there is a lag beyond the canonical function, this could result in contamination of the relatively short 10-s rest blocks with condition blocks. This may not normally cause a significant issue. However, where there is a profound difference in signaling comparing blind and sighted conditions, the least salient blind field conditions may be negatively impacted.

### Plasticity Underlying Blindsight as a Tool for Training

In the past, a difference between the superior collicular response to unseen negatively conditioned faces in GY and healthy controls was attributed either to a difference in the lateralized nature of stimulus presentation, or to a change in collicular functioning following occipital lobe injury (5, 42). Our observation that the superior colliculus responded significantly to traits in the blind but not sighted hemifield of patients, despite identical eccentricities, suggested that the latter explanation was likely to be more important. Neuroplasticity after striate cortex lesions has been observed in non-human primates, resulting in an increase in the number of collicular cells with enhanced response to visual targets (31). This has important implications for the rehabilitation of visual loss in cortical blindness by capitalizing on and further enhancing such changes.

There are other examples of plasticity in the subcortical affective pathways after V1 damage. Tamietto et al. (38) found an almost 10-fold increase in the strength of collicular and amygdala connections in the damaged hemisphere of GY compared to that of his intact hemisphere, and an almost 16-fold increase compared to controls. While fascicle number is disputed as physiologically significant (50, 51), an increase in the fractional anisotropy (FA) is often considered to indicate plasticity, such as following motor learning (52). GY demonstrated a significant increase in FA compared to 10 age-matched controls that was specific to the damaged hemisphere. Together, this suggests that the superior colliculus possesses some degree of plasticity in order to generate new or recovered functionality (such as from early childhood, or evolutionarily) to support blindsight when the primary visual pathway is damaged. If this can be capitalized upon, it may be possible to target this pathway to improve residual vision after brain injury.

## Conclusions

The phenomenon of blindsight is unique as it permits the comprehensive investigation of residual structures and functions, to provide evidence for the underlying structures and pathways (13, 14). In the current study, we showed that subtle and complex facial expressions of trustworthiness and dominance could survive processing when V1 is damaged, and were mediated by the superior colliculus and its intact functional connection with the amygdala. This suggests, as predicted, that the implicit evaluation of social trait shares the same underlying mechanism as affective processing and can occur independent of the primary visual cortex.

## Data Availability Statement

The datasets generated for this study are available on request to the corresponding author.

## Ethics Statement

The studies involving human participants were reviewed and approved by Oxford Research Ethics Committee Ref B08/H0605/156. The patients/participants provided their written informed consent to participate in this study.

## Author Contributions

HB and SA contributed to conceptualization, methodology, analysis, and writing of the manuscript. SA additionally contributed to project administration. MP contributed to analysis. All authors contributed to the article and approved the submitted version.

## Conflict of Interest

The authors declare that the research was conducted in the absence of any commercial or financial relationships that could be construed as a potential conflict of interest.
