# Supplementary figures and images for: The Superior Colliculus and Amygdala Support Evaluation of Face Trait in Blindsight
Source: Front Neurol. 2020 Jul 17;11:769. doi: 10.3389/fneur.2020.00769 (PMC7379153; doi:10.3389/fneur.2020.00769)

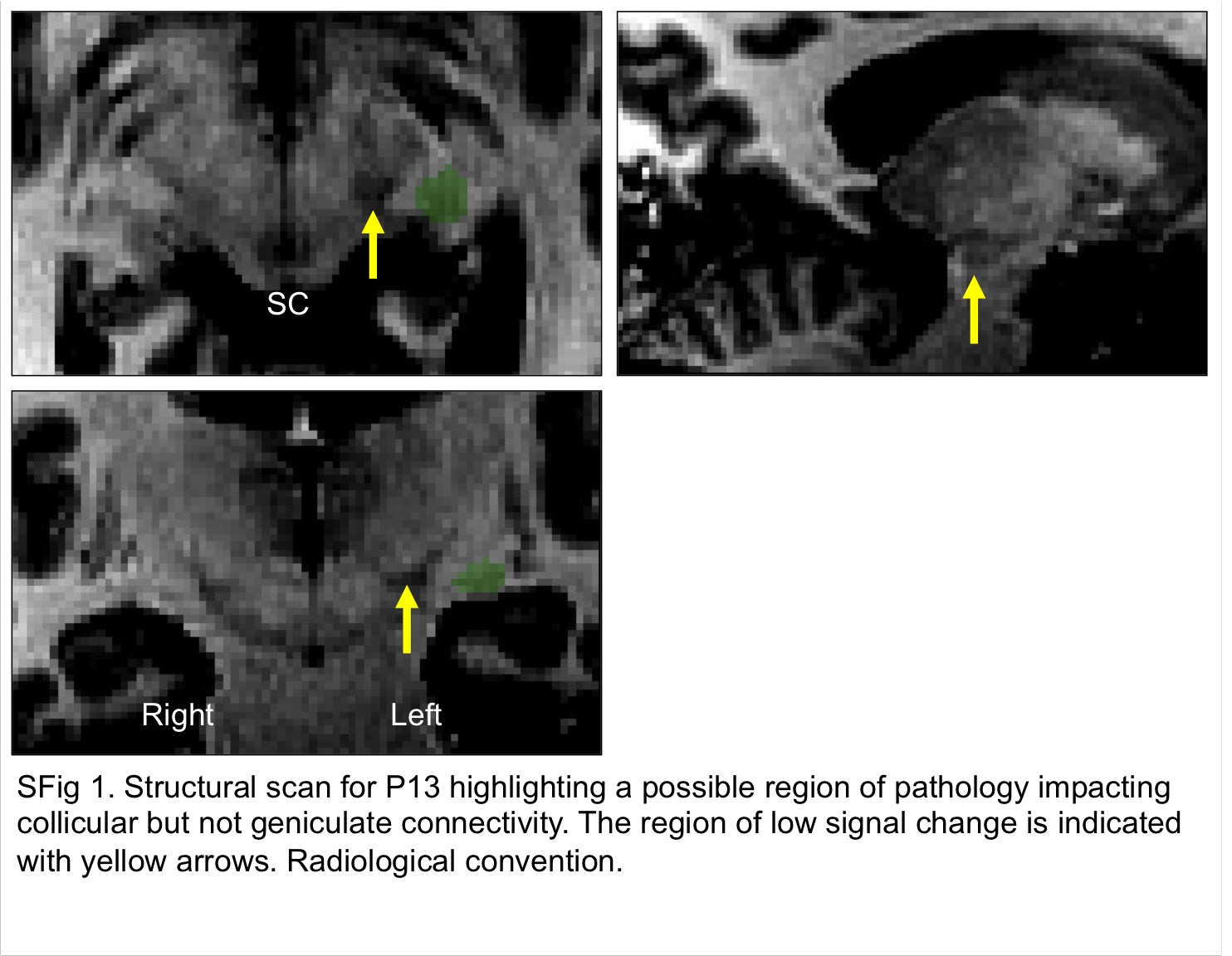

Supplement: Supplementary file 1 [file Image_1.TIFF]
